# “More Spiritual Health Professionals Provide Different Care”: A Qualitative Study in the Field of Mental Health

**DOI:** 10.3390/healthcare11030303

**Published:** 2023-01-19

**Authors:** Rocío de Diego-Cordero, Ángeles C. López-Tarrida, Carmen Linero-Narváez, José María Galán González-Serna

**Affiliations:** 1 Faculty of Nursing, Physiotherapy and Podiatry, University of Seville, 41009 Seville, Spain; 2 Department of Critical Care and Emergency, Hospital Saint John of God Aljarafe, Hospitaller Order of Saint John of God, 41930 Bormujos, Spain; 3 Sant Joan of God Center of Nursing, University of Seville, Health Sciences Teaching and Research Campus San Juan de Dios, 41930 Bormujos, Spain

**Keywords:** mental health, spiritual care, spiritual needs, spirituality, religiosity

## Abstract

In recent years, there has been an exponential increase in studies demonstrating the positive effects of the religiosity/spirituality (R/S) approach on patients’ physical and mental health. In mental health units, patients want professionals to be sensitive to their spiritual and/or religious needs, which is a fundamental aspect of a holistic approach to patients. Qualitative research with an exploratory and descriptive design with an ethnographic-phenomenological approach through in-depth interviews with sixteen professionals from Spanish mental health units. There is no consensus on the definition of spirituality; however, all of them believe that R/S has a positive influence on the health of patients in coping with illness. They believe that their own R/S may interfere with the professional–patient relationship and their attention to spiritual needs. Few professionals address these needs, citing limitations such as lack of time and lack of specific training in spiritual care. More research is needed on the spiritual care of mental health professionals to define the framework, professional training, and associated challenges in the spiritual care of patients.

## 1. Introduction

Although there is no generally accepted concept, spirituality is often seen as a dynamic and inherent dimension of being human, related to the ways in which people (understood as individuals or communities) feel, express, and/or seek meaning, purpose, and transcendence, and the ways in which they connect with self or relate to others, nature, or the sacred [1].

It is this meaning that most separates from religiosity, spirituality being understood as a broader idea. The two concepts are not mutually exclusive and can coincide, overlap, or exist separately [2]. In this way, religion can be defined as the set of beliefs, rules, practices, or rituals related to the transcendent that develops in a community [3].

In recent years, studies addressing the relationship between spirituality and health have increased exponentially [4]. Numerous research studies have shown the positive effects of a religiosity/spirituality (hereafter R/S) approach on the physical and mental health of patients [5]. Spirituality can be developed as a source of strength that promotes quality of life and adaptation to illness [6].

In this context, addressing the spiritual needs of patients and their families in mental health units is of great value. Numerous studies have already confirmed the importance and benefits obtained through spiritual care in these units, showing positive effects on anxiety/depression levels, a lower tendency to suicide or substance use, and improvements in coping with illness [5,7,8,9].

Despite the evidence, few professionals currently include the spiritual dimension in their clinical practice [10]. Due to this situation, there is a deficit in spiritual care, even though most patients want a greater incorporation of the spiritual dimension in medical discussions [11]. Several studies have analyzed the different barriers detected by professionals to including this care, highlighting among them lack of time, lack of training, terminological confusion, and fear of offending the patient [12,13,14].

It should be noted that patients with mental disorders in their acute processes may have alterations in their perceptions, and cases of delusions related to their R/S are frequently described. With all this, adding to the lack of training of professionals and their doubts about the benefits they can obtain, it can be said with the available evidence that the approach to spiritual care in mental health remains a taboo subject [15].

R/S and psychiatry have historically had a complicated relationship, influenced by beliefs, misinterpretations, and the dominance of the biomedical model. They should be seen as allies since spiritual well-being plays a fundamental role in both mental and physical health, with distorted or healthy faith or spirituality having very different effects on patients’ lives. Patients want mental health professionals to respect and be sensitive to their spiritual or religious beliefs and practices, and spiritual care is a fundamental aspect of a holistic approach to the mental health patient [16].

In this context, it becomes evident that more research is needed to understand the qualitative experiences of mental health professionals towards spiritual care to define practice, professional training, and associated challenges in the spiritual care of patients [14].

To fill this gap, this study aims to investigate the perceptions, knowledge, and attitudes of professionals working in mental health units in Spain about the spiritual needs of patients and families and the spiritual care provided during their clinical practice.

## 2. Materials and Methods

### 2.1. Design

A qualitative, exploratory, and descriptive study design with an ethnographic-phenomenological approach was carried out. This study was registered in OSF (identifier: DOI 10.17605/OSF.IO/HQN4P).

This approach is characterized by (a) a conceptual model provided by the researchers; (b) a discrete group or collective perspective; (c) being issue-oriented within a particular setting; (d) using a limited sample of participants who may also have specific knowledge; and (f) occasional participant research [17].

Data collection consisted of in-depth interviews conducted by four researchers (three nurses and one physician) with experience in spiritual care and different publications in the field of spirituality and health, from November 2021 to February 2022.

### 2.2. Sample

Participants working in mental health care units in public and/or private hospitals in Spain were included.

All religious staff in health centers, health professionals working in other services or units, as well as non-health professionals, were excluded.

### 2.3. Data Collection

The sample was collected through non-probability sampling, firstly, by convenience, since the sample of professionals from a specific field of the population of interest is chosen without using statistical criteria for their selection by using the professional and personal contacts of the researchers.

To increase the number of participants, a snowball sampling procedure was also used, with the aim of achieving a more diverse sample in terms of professional positions, experience, and age.

The researchers contacted the participants by telephone, applying the eligibility criteria. Interviews were conducted by the four researchers, face-to-face, lasting approximately 40–50 min, which were subsequently recorded and transcribed for analysis. Data collection continued until saturation was reached.

### 2.4. Instrument

An interview script with open-ended questions divided into five sections was used. This interview guide is presented in Appendix A. The first two sections cover the socio-demographic characteristics and the characteristics of the spiritual dimension of the participants. The third section deals with the views, attitudes, and barriers to addressing patients’ R/S in clinical practice and the influence of R/S on patients’ health; the fourth section assesses the R/S care of the mental health patient; and the fifth section assesses professionals’ views on academic training and teaching.

### 2.5. Data Analysis

The interviews were transcribed verbatim and read in depth. Categorization based on the findings was carried out using Nvivo Version 12 software, focusing on persistent or emerging concepts and similarities/differences in participants’ statements and comparisons. These coded data were examined individually and then compared with other participants’ data to develop categories.

To guarantee the quality of the analysis, data triangulation was carried out, including participants with different socio-demographic characteristics, and the triangulation of data analysis through the four researchers. For their identification, the discourses were denoted as follows: participant number, gender, age, and professional category.

### 2.6. Validity

This research followed the EQUATOR Research Checklist, specifically the guide used to evaluate qualitative articles: Consolidated criteria for reporting qualitative research (COREQ): a 32-item checklist for interviews and focus groups [18]. These reporting guidelines are presented in Table A2.

Methods used to enhance the validity of this qualitative study included triangulation of data (including participants with different socio-demographic characteristics) and triangulation of data analysis by different researchers.

### 2.7. Ethical Considerations

The study received acceptance from participants who were invited to participate voluntarily by the researchers, receiving prior information about the project and signing the informed consent for their participation. The information included the possible risks of the study and explanations about the right to refuse to answer questions and/or to terminate the interviews at any time. In addition, participants were informed that the interviews would be audio-recorded, quoted anonymously in publications, and that all personally identifiable information would be withheld. Furthermore, verbal consent was obtained from participants. The study was approved by the Research Ethics Committee of Andalusia, Spain (code:0731-N-19).

## 3. Results

### 3.1. Sociodemographic Characteristics of the Sample

The sample consisted of a total of 16 professionals: 8 nurses, 3 psychiatrists, 1 teacher, and 4 psychologists. Of these, 56.25% were women and 43.75% were men, with an average age of 45.9 years (range 24 to 63 years), all of them Spanish, and with an average of 20.25 years of professional experience.

Nine participants work in the mental health services of the “XX” to keep the paper blind (56.25%) in Seville (43.75%), Malaga (37.5%), and Madrid (18.75%). Regarding their spiritual and religious beliefs, 50% of the total sample defined themselves as spiritual and religious (all of them Catholics), 37.5% as spiritual but not religious, and 12.5% as religious but not spiritual.

Of the professionals in the sample, 93.75% received postgraduate training; however, only 25% of these received specific training related to the approach to spirituality. The socio-demographic characteristics of the sample are presented in Table 1.

The analysis yielded three main themes that are reflected in the following categories: “Addressing spirituality in clinical practice and its influence on health”, “Barriers to providing spiritual care in mental health”, and “Training in spiritual care for health professionals”.

### 3.2. Addresing Spirituality in Clinical Practice Its Influence on Health

The entire sample has heard the term spiritual health at some time; however, there is no consensus on the definition of spirituality.

Some of the participants would not even know how to describe it (*P-9*, *male*, *63 years old*, *nurse:* “*well*, *frankly I don’t know*”*; P-5*, *woman*, *56 years old*, *nurse:* “*well*, *I really wouldn’t know how to explain it*”), however, most of them related it to people’s values or beliefs (*P-12*, *man*, *24 years old*, *nurse:* “*I understand it a little bit in terms of people’s values or beliefs*”; “*I understand it a bit directed to the patient’s situation at the moment they are in and how all that affects their values*, *their beliefs*, *their training*, *their education*, *all that that implies*”), well-being and self-care (*P-3*, *female*, *45 years old*, *nurse:* “*the patient’s wellbeing according to their religious or spiritual beliefs*”; *P-7*, *female*, *28 years old*, *nurse:* “*I think it is the wellbeing of beliefs*”), state in which one feels good with oneself (*P-1*, *woman*, *32 years old*, *psychologist:* “*I understand that it is a state in which one feels good with oneself*, *where one finds a state of acceptance and tranquility*, *of not judging oneself*, *for me that is the spiritual part*...*to look for the meaning that it has for each one and to strengthen that in each person*”), or they identified it as another need of the human being (*P-10*, *man*, *50 years old*, *nurse:* “*as another need of the person*, *when they come here to be admitted*, *the same as there is a physiological need for psychological health*, *there is also the need for transcendence*”).

All professionals believe that R/S influences the health of patients in some way, relating it in a positive way to coping with the illness:

*P-4, male, 50 years old, teacher: “I think so, above all in the acceptance of the illness...with the illness you have to reconcile yourself, with the illness you have to pray and if the person is a believer or, even if they are not, well I think that spiritual help is going to help them to cope with that illness and that increase in the ordeal of suffering that person has at that moment”.*

*P-1, woman, 32 years old, psychologist: “Well, and sometimes there are those who understand and accept their illness with religion, so I think that religion and spirituality do help them to cope with mental illness many times”.*

Furthermore, they believe that their own R/S may interfere with the professional-patient relationship and willingness to address their spiritual needs, stating that those who care for their own spirituality establish and provide better spiritual care:

*P-14, male, 52, psychiatrist: “The spirituality of the health professional influences the therapeutic relationship, I am convinced, the more spiritual health professionals provide different care... I believe that a more spiritual person is going to be more receptive, let’s say to the spiritual demands of the patient”.*

*P-5, female, 56, nurse: “We have colleagues who are very spiritual and for example, they have a way of implanting, of communicating or seeing things in a different way”.*

Despite this, they do not feel the desire to address the spiritual needs of their patients, doing so on an ad hoc basis when they detect spiritual distress.

*P-3, female, 45, nurse: “I don’t feel the desire, but if I detect it or if the subject is brought up, I address it openly”.*

*P-11, female, 55, psychologist: “When I detect a very clear spiritual need, I usually turn to the pastoral worker to address it”.*

Moreover, none of them has considered addressing the spirituality of the relatives of mental health patients, although many of them consider that it would be very interesting and that they should improve this aspect:

*P-12, male, 24, nurse: “I haven’t thought about it, but I do think it is an area that needs to be worked on and explored a lot”.*

### 3.3. Barriers to Providing Spiritual Mental Health Care

During the interviews, most of the professionals (n = 14) reported that they felt difficulties or barriers that discouraged them from discussing R/S with their patients, stating also that there are possibilities to improve the spiritual care of mental health patients, relating the latter to an improvement in the specific training of the professionals working in these units. Among the most frequently mentioned barriers are lack of time, related to work overload and lack of staff, and lack of training.

*P-2, man, 57 years old, psychiatrist: “For me the main barrier is time, and there are also barriers that are a little more personal, that is, whether or not the person feels able to enter into this type of approach.*

*P-7, female, 28, nurse: “If we were given the possibility to spend more time with patients, to dedicate more time to them, and, well, to provide more professionals”.*

*P-1, female, 32, psychologist: “Time, means and people, because sometimes I think that spiritual activities need time not only to try them out, but also to think about them and approach them”.*

Other difficulties identified were the fear of not knowing how to control the situation (*P-12*, *woman*, *45 years old*, *nurse:* “*I see it as a taboo area*, *which is respected but better to leave it*... *unless they address it but without me entering*”), the existing stigma when addressing the subject, (*P-8*, *man*, *45 years old*, *nurse:* “*There is a lot of stigma*, *even among health professionals*, *mental health patients are not given the same treatment or credibility*, *with their mental illness weighing more heavily than other aspects of their health*”), the difficulty in identifying the need to address this dimension (*P-10*, *man*, *50 years old*, *nurse:* “*when a problem of these characteristics is presented to you*, *look the other way*... *and this may be present in some professionals in the sector because they are issues that perhaps make us uncomfortable and we prefer to say well*.... *this for another professional in another field and I’ll forget about it*”), or the lack of professional experience (*P-4*, *man*, *50 years old*, *teacher:* “*the truth is that you find inexperienced people who come to work in the centers who have not being yet ingrained the concept that a person’s wellbeing lies in physical*, *psychological*, *social and spiritual development*”).

Only two of the participants stated that there are currently no barriers or difficulties in providing spiritual care to mental health patients.

When asked if they would know how to improve or eliminate these barriers or difficulties, a minority stated that they would not know how to do so, and the rest of them highlighted, as possible measures, the training of professionals (*P-15*, *woman*, *45 years old*, *psychologist:* “*training professionals so that they can explore and help patients to develop their spiritual dimension*), and an increase in resources or the transition from the classic biological model so deeply rooted in our system to a holistic model that considers the psychological, social or spiritual sphere of the patient (*P-16*, *woman*, *40 years old*, *nurse: well*....*to base ourselves on the theory of holistic care*. *We are biological*, *spiritual*, *emotional*, *and psychological beings*, *I mean*... *everything is important*”).

Most of them consider that the resources that exist to address the spirituality of patients are scarce or unknown to the professionals (*P-5*, *woman*, *56 years old*, *nurse:* “*because if we don’t even know what resources I can access or how*, *we are probably using many tools without knowing that we are using them or not using others that we have available*”), limiting themselves to the possibility of contacting a chaplain on those occasions when the patient or their relatives request it (*P-16*, *woman*, *40 years old*, *nurse:* “*The resources that exist*...*well if they are rather scarce or are not known by society*...*and the devices that can be useful well*...*in hospitals there is the attention of the chaplain or masses*”).

### 3.4. Spiritual Care Training for Health Professionals

The lack of preparedness to address religious/spiritual aspects of mental health patients is a common statement shared by the professionals in the sample:

*P-5, female, 56 years old, nurse: “Failed, a 3 or 4. We are trained on many things, on how to administer medication, for that we are always much better trained. Very little training is dedicated to these things and for us I think it is fundamental in Mental Health”.*

The majority of professionals (n = 14) consider it useful for spiritual/religious care to acquire a greater value within university education, stating that there is no need for a specific subject, but rather for it to be a cross-cutting subject during the degree (*P-7*, *woman*, *28 years old*, *nurse:* “*I think it could be a cross-cutting subject*, *it could fit into many subjects and be remembered throughout the years*”).

They also state that training in spiritual care is very important for their work in mental health:

*P-16, female, 40, nurse: “Well, it is very important, as I said before, the human being is everything, bio-psycho-social and spiritual, and there are many things that have not been taken into account, and there are many spiritual crises, and we are not aware that our users have them”.*

## 4. Discussion

This study aimed to describe the perceptions, knowledge, and attitudes of mental health care professionals regarding the spiritual needs of patients and their families about the spiritual care they provide in their clinical practice.

The results indicate that there is no consensus among the participants on the definition of spirituality, mainly due to terminological confusion with the concept of religiosity; however, they all believe that R/S influences patients’ health in some way, relating it positively to coping with illness. Furthermore, they believe that their own R/S may interfere with the professional–patient relationship and their willingness to address their spiritual needs. Yet, few professionals regularly address the spiritual needs of their patients. Most professionals reported that they experience difficulties or barriers that discourage them from discussing R/S with their patients, especially a lack of time and specific training.

Regarding the approach to spirituality in clinical practice and its influence on health, despite the diverse religious affiliations of the participants, all the professionals interviewed believed that R/S influences patients’ health in some way, relating it positively to coping with mental illness. Several studies reflect this finding, showing a significant correlation between patients’ approaches to spirituality and an improvement in coping with illness and in physical, social, psychological, and spiritual quality of life. The study by Camargos et al., 2015 [19], conducted in Brazil with a sample of 1050 participants (525 healthcare professionals and 525 cancer patients), reveals that 94.1% of patients considered it important for healthcare professionals to ask them about their spiritual beliefs, and 98.3% of healthcare professionals agreed that spiritual care was necessary for patients. They also compared levels of quality of life between patients receiving only curative treatment and those receiving palliative care that included a spiritual approach, with the latter having higher levels of social and psychological quality of life. Similarly, a meta-analysis by Xing et al., 2018 [20] of seven studies with 1134 patients (575 in the intervention group and 559 in the control group), reported the effect of spiritual interventions on the spiritual well-being of cancer patients after treatment, indicating a statistically significant difference in the effects obtained on the well-being of patients included in the intervention group. The study by Jongkind et al., 2019 [21], in patients with depression, reveals that those patients who use religion as a method of coping with their illness have lower rates of suicidal ideation and better control of their mental illness/disorder/pathology, compared to those patients who are neither religious nor spiritual.

On the other hand, participants believe that their own R/S may interfere with the professional–patient relationship and their willingness to address their spiritual needs, stating that those who take care of their own spirituality establish and provide better spiritual care. This finding is described by other studies, in which those professionals who have a more developed spirituality spend more time addressing the spiritual needs of their patients, establishing a symmetrical professional–patient relationship, and offering greater opportunities for dialogue about their spiritual interests [22,23,24,25]. In a study of nurses working in a mental health unit, nurses described their own spiritual/religious characteristics as significant factors for spiritual care, with those nurses who saw themselves as ‘spiritual and religious’ providing spiritual care more frequently than those who saw themselves as ‘spiritual but not religious’ [26].

The results of our study showed that most professionals are in favor of incorporating the spiritual dimension into clinical practice; however, few professionals currently include spiritual care in their daily care, mostly on those occasions when they detect spiritual distress. Despite increasing research in the field of spirituality and recognition by professionals of spiritual practices, little attention is paid to the spiritual approach in clinical practice or professional training due to the entrenchment of the biomedical model in our health care system [27]. As a result, there is a deficit in spiritual care, even though most patients desire greater incorporation of the spiritual dimension in encounters with healthcare professionals [11,28,29].

About barriers to providing spiritual care in mental health, fourteen of the sixteen participants stated that they experience difficulties that discourage them from discussing R/S with their patients, including lack of time, lack of training, fear of not knowing how to manage the situation, stigma attached to addressing the issue, and difficulty in identifying the need. Most participants reported a lack of time as one of the main perceived barriers to spiritual care. This finding is identified in several studies [13,30,31,32]. Notable among these is the study by Chen et al., 2017 [33], in which eighteen nurses in Singapore were interviewed, all of them recognizing a lack of time as the main barrier to addressing the spiritual needs of their patients. Most of them felt that physical medical care is prioritized over psychosocial care, expressing that spiritual care is only provided at times when “there was extra time”.

A more recent study, conducted during the pandemic by COVID-19 on a sample of 19 nurses working in intensive care units and emergency departments in Spain, showed that nurses were responsible for providing spiritual care to their patients [34]. In general, they believed that R/S was an essential aspect of helping patients cope with illness in these units; however, they did not feel empowered to provide adequate spiritual care in these crisis situations. As in our study, nurses cited several barriers, such as lack of time and training [35].

Looking at the other barriers, in a study of a sample of 279 clinical social workers, almost half of them stated that they waited for patients to initiate dialogue related to their religious and/or spiritual interests for fear of offending the patient, because they felt uncomfortable, or because they considered addressing this need a taboo subject or belonging to the patient’s most intimate sphere [36].

Most participants felt that the resources that exist to address patients’ spirituality are scarce or unknown to professionals, being limited to the possibility of contacting a chaplain on those occasions when the patient or family members request it. The study by Siler et al., 2019 [37] in the USA, in a sample of nineteen healthcare professionals, described how participants most frequently turned to the chaplain to offer spiritual support to patients, particularly when they did not feel comfortable in the clinical encounter, did not know how to address the spiritual dimension, or felt that they were not comfortable with the spiritual dimension. Similarly, another study described how practitioners turned to the chaplain when patients or family members brought up uncomfortable religious or spiritual issues [38].

In line with the findings described above, when asked whether they would know how to improve or eliminate these barriers or difficulties, a minority of the professionals interviewed stated that they would not know how to do so, while the rest highlighted as possible measures specific training, an increase in resources, or a shift from the classic biological model so deeply rooted in our system to a comprehensive model that considers the psychological, social, and spiritual sphere of the patient. The struggle for the humanization of care in health centers is currently on the rise, and it is Jean Watson who contributes this more humanized vision in his Theory of Human Care, advocating integral patient care: “Faced with the risk of dehumanization in patient care, due to the great administrative restructuring of most health care systems in the world, it is necessary for nursing professionals to rescue the human, spiritual and transpersonal aspect in clinical, administrative, educational and research practice” [39].

Regarding training in spiritual care for health professionals, another of the main issues referred to by the interviewees is the lack of training as the main limitation for an adequate approach to the spiritual needs of patients in mental health units. As referred to in research carried out and published on the subject, this is the main gap reported by health professionals, regardless of the clinical context in which the studies have been conducted [23,40].

In turn, there is a range of research highlighting patients’ desires for their caregivers to talk to them about their spiritual and/or religious beliefs [11,28,29]. Following this line, the study by Kichenadasse et al., 2017 [41], in a sample of 69 physicians, described that most of the professionals interviewed had encountered patients who expressed spiritual needs during clinical consultations; however, only a minority of them perceived that they could meet the spiritual needs of their patients, stating the lack of training as the main barrier, as only a small percentage stated that they had received education in this regard during their professional development. Furthermore, different studies highlight that nurses, compared to physicians, tend to be more sensitive and more willing to address the spiritual needs of patients, related to the higher percentage of nurses who have received university training in spiritual care compared to physicians [42,43]. Different studies point to the considerable benefits of incorporating R/S subjects in the curricula; future professionals feel better prepared and more comfortable to approach and provide spiritual care, improving the assessment and holistic care of patients. Another study conducted on Spanish students identified how many of them felt underprepared to address the spiritual needs of patients, believing that universities are not providing enough training on spiritual care in clinical practice. In addition, nursing students tended to believe more in the influence of R/S on patients’ health and the appropriateness of addressing religious and spiritual issues [10,35].

A clinical trial provided training in spiritual care to nurses from different services in a Rotterdam hospital, showing that after the training, the patients cared for were more supportive and responsive to their spiritual needs. There were also significant changes in nurses’ attitudes and knowledge, as well as improvements in clinical practice, when documenting and addressing patients’ spiritual needs and considering referrals and help from other professionals [44].

Adequate training provides tools for spiritual care for both practitioner and patient and has been shown to be of paramount importance [11,45]. Training in spiritual care is a fact of great relevance for providing adequate spiritual care, as this training has a positive impact on holistic and humanized care [20,46,47,48].

The inclusion of spiritual care in academic curricula, in a regulated way and adjusted to the training of the future professional, affects the clinical relationship and decision making in practice [22]. Previous proposals confirm the existing interest in improving this circumstance [3,49,50].

## 5. Conclusions

The spiritual and/or religious sphere is considered an essential dimension of patient care in mental health units, as observed in the opinions and perceptions of the Spanish professionals included in this study. However, lack of specific training and lack of time are important barriers detected by professionals for the provision of spiritual care in mental health units.

Lack of adequate training in spiritual care has been identified as an important predictor in addressing the spiritual dimension, as although most professionals encounter patients with spiritual needs, only a minority perceive that they are able to meet them due to lack of training during their professional apprenticeship.

Further research on spiritual care is needed to understand the qualitative experiences of mental health professionals towards spiritual care to define practice, practitioner training, and associated challenges in the spiritual care of patients.

### Relevance to Clinical Practice

Therefore, conducting future interventions focused on providing mental health unit professionals with the skills and support to improve their ability to integrate spiritual care into clinical care will improve health outcomes.

For this, more research is necessary to know the true current situation in relation to the spiritual care currently provided by professionals in the field of mental health and thus be able to implement effective actions in this field.

What does this paper contribute to the wider global clinical community?

- There is a lack of attention to mental health in terms of spirituality, even though its correlation with mental health and its potential benefits have been widely demonstrated.
- The spiritual dimension of care remains a challenging task for the modern health system.
- More research is needed around spirituality and mental health to push the uncharted limits and give visibility to its multiple functionalities.

## Figures and Tables

**Table 1:** Socio-demographic characteristics of the sample.

Gender		
Variable	Absolute Value	Percentage
Female	9	56.25%
Male	7	43.75%
**Ethnicity**		
**Variable**	**Absolute Value**	**Percentage**
White/European (Spanish)	16	100.00%
**Marital Status**		
**Variable**	**Absolute Value**	**Percentage**
Single	5	31.25%
Married	9	56.25%
Separated	2	12.50%
**City of residence**		
**Variable**	**Absolute Value**	**Percentage**
Seville	7	43.75%
Málaga	6	37.50%
Madrid	3	18.75%
**University degree**		
**Variable**	**Absolute Value**	**Percentage**
Nurses	8	50.00%
Psychiatrists	3	18.75%
Psychologists	4	25.00%
Teachers	1	6.25%
**Postgraduate training**		
**Variable**	**Absolute Value**	**Percentage**
Yes, R/S related	4	25.00%
Yes, not related to R/S	11	68.75%
No	1	6.25%
**Hospital service**		
**Variable**	**Absolute Value**	**Percentage**
Saint John of God Hospitaller Order (Mental health unit)	9	56.25%
Therapeutic Community Hospital del Tomillar Intensive Community	6	37.50%
Mental Health Programme Hospital el Tomillar	1	6.25%
**Religious affiliation**		
**Variable**	**Absolute Value**	**Percentage**
Spiritual and Religious	8	50.00%
Spiritual, but not religious	6	37.50%
Religious, but not spiritual	2	12.50%
Neither spiritual nor religious	0	0.00%

Average age: 45.93 years; average number of years of professional experience: 20.25 years.

## Data Availability

The data presented in this study are available on request from the corresponding author.

## Appendix A

**Table A1:** Interview guide.

Socio-Demographic Data
What gender do you identify with?
How old are you?
What is your nationality?
What is your marital status?
What was your place of birth?
What is your current city of residence?
What is your university degree?
How many years of professional experience do you have in Mental Health?
In which mental health unit do you currently work?
Postgraduate training: Do you have any training in mental health? Do you have any training in spirituality/religion? Spirituality/religion: Which one?
Dimension of religiosity
How do you feel: religious, spiritual, spiritual and religious, or would not know how to answer? Describe if you can.
Could you describe your religious affiliation if you have one?
Clinical practice and spirituality
Have you ever heard the term spiritual health and what do you understand by it?
Do you think religion/spirituality influences patients’ health or coping with illness in any way? If so, how do you think it influences it?
In your opinion, does the spirituality/religiosity of health professionals interfere with the professional-patient relationship? In what way does it influence it?
Do you feel a desire to address the issue of faith/spirituality with patients?
Have you ever asked your patients about religion/spirituality? * (If “Yes”: How often do you usually ask this question, when or in what situations do you usually address this question? religious or spiritual aspects that characterized them?
How do you think you can provide spiritual care in your daily activity?
Do you experience difficulties or barriers that discourage you from discussing religion/spirituality with your patients? religion/spirituality with your patients? Which ones?
Religion/spirituality in the approach to the mental health patient
In your daily practice, do you address or value the spiritual dimension of your patients?
What is the role of your own spiritual or religious beliefs in assessing or approaching the patient?
How do you perceive that the individual’s beliefs influence the course of their illness?
Can you tell me if your patients use their own spiritual/religious beliefs as a way of coping with their illness?
Have you ever avoided discussing religious or spiritual issues with your patients? Could you describe why?
Have you considered assessing and addressing the spiritual/religious needs of your patients’ families? If so, how have you done so?
How do you think spiritual care could be improved, are there resources or some kind of help that could be useful?
Academic training in spirituality
Do you think it would be useful for these aspects and spiritual/religious care to have more value within university education? Where: in undergraduate studies? in specific subjects? in a transversal way? in postgraduate studies: master’s degree or expert? Do you know anything?
How important might this training be for your work in the mental health field? Why?

**Table A2:** Consolidated criteria for reporting qualitative studies (COREQ): 32-item checklist.

No	Item	Guide Questions/Description	Response
Domain 1: Research team and reflexivity			
Personal Characteristics			
1.	Interviewer/facilitator	Which author/s conducted the interview or focus group?	All the interviews were conducted by the four authors (RDC, ACLT, CLN).
2.	Credentials	What were the researcher’s credentials? e.g., PhD, MD	RDC and JGGS were Phd. ACLT and CLN were MsC.
3.	Occupation	What was their occupation at the time of the study?	RDC and JGGS were researchers, ACLT was physician, and CLN was nurse.
4.	Gender	Was the researcher male or female?	RDC, ACLT and CLN were female. JGGS was a man.
5.	Experience and training	What experience or training did the researcher have?	All researchers had experience in carrying out qualitative research and the have been trained to conduct interviews.
Relationship with participants			
6.	Relationship established	Was a relationship established prior to study commencement?	No, there wasn’t.
7.	Participant knowledge of the interviewer	What did the participants know about the researcher? e.g., personal goals, reasons for doing the research	Name, occupation, reason for doing the research.
8.	Interviewer characteristics	What characteristics were reported about the interviewer/facilitator? e.g., bias, assumptions, reasons, and interests in the research topic	Name, occupation, reason for doing the research.
**Domain 2: Study design**			
Theoretical framework			
9.	Methodological orientation and Theory	What methodological orientation was stated to underpin the study? e.g., grounded theory, discourse analysis, ethnography, phenomenology, content analysis	Phenomenological and ethnographic approach with a discourse and content analysis.
Participant selection			
10.	Sampling	How were participants selected? e.g., purposive, convenience, consecutive, snowball	Snowballing For convenience
11.	Method of approach	How were participants approached? e.g., face-to-face, telephone, mail, email	Telephone and face-to-face
12.	Sample size	How many participants were in the study?	16 mental health professionals, 9 women and 7 men, aged between 24 and 63 years.
13.	Non-participation	How many people refused to participate or dropped out? Reasons?	No participant
Setting			
14.	Setting of data collection	Where was the data collected? e.g., home, clinic, workplace	The interviews were carried out face-to-face in different places.
15.	Presence of non- participants	Was anyone else present besides the participants and researchers?	No, it wasn’t.
16.	Description of sample	What are the important characteristics of the sample? e.g., demographic data, date	Mental health professionals, workers in mental health units, Spain.
Data collection			
17.	Interview guide	Were questions, prompts, guides provided by the authors? Was it pilot tested?	Yes, they were. Yes, it was.
18.	Repeat interviews	Were repeat inter views carried out? If yes, how many?	No, they weren’t
19.	Audio/visual recording	Did the research use audio or visual recording to collect the data?	Audio recording
20.	Field notes	Were field notes made during and/or after the interview or focus group?	Yes, field notes.
21.	Duration	What was the duration of the inter views or focus group?	About 40–50 min
22.	Data saturation	Was data saturation discussed?	Yes, it was
23.	Transcripts returned	Were transcripts returned to participants for comment and/or correction?	No, it wasn’t.
**Doman 3: Analysis and findings**			
Data analysis			
24.	Number of data coders	How many data coders coded the data?	Two (CLN and RDC).
25.	Description of the coding tree	Did authors provide a description of the coding tree?	Yes, we did.
26.	Derivation of themes	Were themes identified in advance or derived from the data?	Themes were derived using both methods
27.	Software	What software, if applicable, was used to manage the data?	Nvivo Version 12
28.	Participant checking	Did participants provide feedback on the findings?	Yes, they did.
Reporting			
29.	Quotations presented	Were participant quotations presented to illustrate the themes/findings? Was each quotation identified? e.g., participant number	Yes, there were/Yes, there was
30.	Data and findings consistent	Was there consistency between the data presented and the findings?	Yes, there was.
31.	Clarity of major themes	Were major themes clearly presented in the findings?	Yes, they were.
32.	Clarity of minor themes	Is there a description of diverse cases or discussion of minor themes?	Yes, there is.

**Font:** Tong, A. Sainsbury, P., and Craig, J. (2007). Consolidated criteria for reporting qualitative research (COREQ): A 32-item checklist for interviews and focus group. International. Journal Qualitative. Health Care 19: 349–357.
